# Evaluation of day one embryo quality and IVF outcome – a comparison of two scoring systems

**DOI:** 10.1186/1477-7827-7-9

**Published:** 2009-02-03

**Authors:** Jana Brezinova, Ivana Oborna, Magda Svobodova, Helena Fingerova

**Affiliations:** 1 Dept. of Obstetrics and Gynaecology, Faculty of Medicine, Palacky University of Olomouc, Olomouc, Czech Republic

## Abstract

**Background:**

The aim of our retrospective study was to compare the clinical usefulness of two non-invasive embryo scoring systems based either on a simplified pronuclear morphology of the zygote or on early cleavage rate, as well as their combination, for the selection of embryos with the best implantation potential in embryo transfer (ET).

**Methods:**

Over a period of five years, the quality of 2708 embryos from 364 IVF cycles in women under the age of 39 years was assessed using these scoring systems in a university assisted reproduction centre. ET was always performed on day 3 of cultivation. The outcome of ETs of 702 embryos scored in the respective systems or their combination was retrospectively analyzed in terms of biochemical (bPR) and clinical pregnancy rates (cPR) and implantation rate (IR). Mann-Whitney U test and t-test for differences between relative values were used, p < 0.05 was considered statistically significant.

**Results:**

There was no difference in outcome parameters in 109 cycles where only Pattern "0" zygotes, according to our simplified pronuclear morphology classification, were transferred and 140 cycles where only "other" pattern zygotes were transferred, regardless of their cleavage rate. On the contrary, significantly greater cPR and IR (p = 0.003 and p = 0.006, respectively) were achieved in 120 cycles where only early cleavage (EC) embryos were transferred compared with 152 cycles where only non early cleavage (NEC) embryos were transferred regardless of their pronuclear morphology. The best outcome in terms of cPR (56%) and IR (43%) was found in 50 cycles when Pattern "0" and EC embryos only were used for transfer.

**Conclusion:**

The results indicate that early cleavage is a better independent marker of implantation potential than zygote morphology. The best outcome can be achieved if both embryo scoring systems are used jointly and the embryo is classified as EC and Pattern "0".

## Background

Steady improvements to the procedures used in assisted reproduction (AR) have made it possible to retain acceptable success rates with fewer transferred embryos, thus reducing the risk of multiple pregnancies. As the ultimate goal is to achieve successful implantation of a single high quality embryo, much effort has been invested in the search for and validation of reliable non-invasive techniques for assessing embryo quality.

Embryos for embryo transfer (ET) are routinely selected after 2 to 5 days of cultivation using one of a several embryo quality scoring systems [1-3]. Embryo quality is generally determined by cleavage rate, regularity of blastomeres and a low degree of fragmentation. Originally, embryos for transfer were chosen on the basis of their cleavage stage morphology, such as the number and equality of blastomeres, or the presence and grade of fragmentation [1,2]. A non-invasive embryo quality scoring system based on the timing of the first cleavage was introduced by Shoukir et al. [4] and Sakkas et al. [5]. Zygotes which reached the first mitotic division between 25 and 27 hours after insemination, called Early Cleavage (EC) embryos, exhibited more than twice the pregnancy rate and three times the implantation rate compared to non-EC (NEC) embryos.

Meanwhile Tesarik and Greco [6] reported that IVF outcome could be predicted from morphological examination of human zygotes. They classified zygotes into 6 different patterns (0 – 5) based on size and on the number and distribution of nucleoli or their precursors. This system is somewhat impractical for routine use, particularly in a busy IVF laboratory, because it has a very detailed classification and therefore is too time consuming. Consequently, various simplified zygote scoring systems emerged after this publication, using either the number and position of nucleolar precursor bodies (NPB) or nucleoli, or the alignment of NPB which was classified as either polarized or non-polarized [7,8].

There is still no consensus on which of these scoring systems best predicts pregnancy outcome, though several studies including our preliminary ones have demonstrated the benefits of introducing the EC [9-16] or a combination of EC and pronuclear morphology scoring systems [3,17] into clinical practice. Our retrospective study aims to confirm these observations. The outcome parameters evaluated include biochemical pregnancy rate (bPR), clinical pregnancy rate (cPR) and implantation rate (IR) for each of the scoring systems used for the selection of embryos for ET.

## Methods

### Stimulation protocols

Evaluation included 364 IVF cycles in women under the age of 39 at our infertility centre between the years of 2004 to 2006. The main causes of infertility of the couples were male factor (39% of cases) followed by tubal factor (35% of cases), either alone or in combination with other factors. The majority of patients (n = 292) underwent a long follicular depot stimulation protocol. GnRH agonists were applied on Day 1 or 2 of the menstrual cycle, followed by an individualized stimulation with recombinant FSH (rFSH) after achieving down-regulation. Oocyte maturation by hCG was induced when at least 1 follicle reached 20 mm diameter and at least two others were larger than 17 mm. In the remaining 72 patients the stimulation protocol with 100 mg of clomiphene citrate per day from the 3^rd ^to the 7^th ^day of the cycle and hMG or rFSH administration according to individual response in follicular development were used. When two or more follicles reached a diameter of ≥ 18 mm, final oocyte maturation was induced by hCG. In both protocols oocytes were retrieved 36 h later by ultrasound-guided ovum pick-up. Oocytes were fertilized by conventional IVF (n = 96), ICSI (n = 239) or mixed IVF/ICSI (n = 29). Embryo transfer was performed on day 3 of cultivation. Since 2004, the evaluation of reactive oxygen species (ROS) production in ejaculate by the chemiluminescence method [18] has been included in the routine semen evaluation. None of the subjects who entered the study after introducing ROS measurement had excessive ROS levels. Oocytes and embryos were cultured in commercial IVF media (Vitrolife, Sweden).

### Pronuclear morphology scoring system

**Figure 1:**
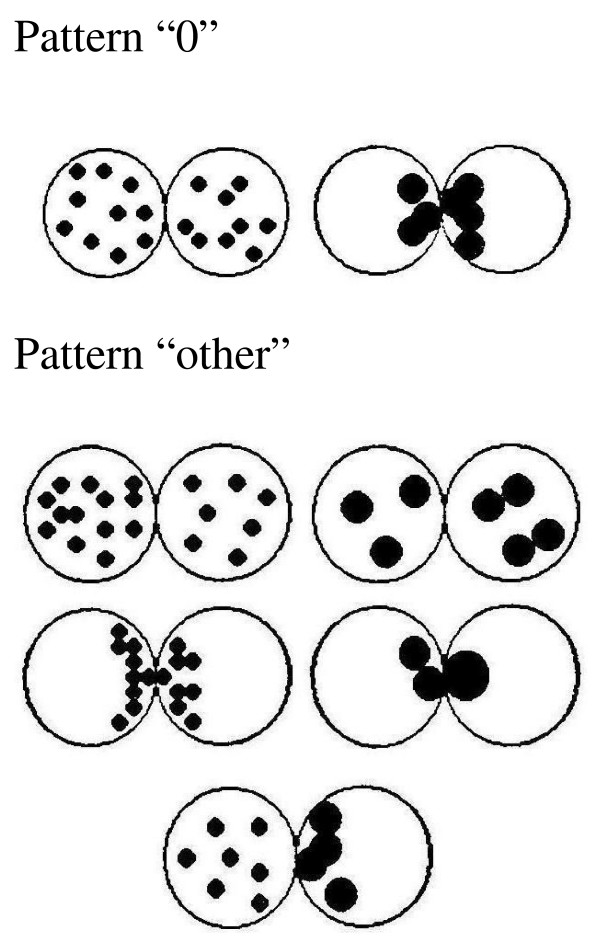
**Simplified classification of pronuclear morphology originally published by Tesarik and Grecco **[6]. **Pattern "0"**: Zygotes exhibiting the same number of small nucleolar precursor bodies (NPB) evenly distributed in the nucleus or large NPB with polarized distribution between the two pronuclei. **Pattern "other": **All other non-symmetrical alignments of the NPBs including different numbers of NBPs.

Evaluation of pronuclear morphology of the zygotes was performed between 16 to 20 hours after insemination/ICSI following [6] but using our own simplified classification. Zygotes exhibiting same number of small nucleolar precursors bodies (NPB) evenly distributed in the nucleus or large NPB with polarized distribution between the two pronuclei were included under the pattern "0". All the other non symmetrical alignments of NPB were classified as pattern "other" (Fig 1). Pattern "0" embryos and "other" embryos were cultivated separately.

### Early cleavage scoring system

The first mitotic division was checked between 23 – 27 hours after insemination or ICSI. Embryos which reached the two cell stage at this interval were classified as EC embryos. Embryos with intact nuclei or nuclear membrane breakdown were classified as No Early Cleavage (NEC) embryos. EC and NEC embryos were cultivated separately.

### Daily routine embryo quality assessment for selection of embryos for ET

Embryos for transfer were primarily selected according to their routine embryo quality assessment, e.g. number of blastomeres, their equality, presence and grade of fragmentation on days 2 and 3 of cultivation and transferred on day 3. If a large number of embryos with the same morphologic quality were obtained in individual patients, the EC and/or pattern "0" embryos were preferentially transferred.

### Outcome parameters

Biochemical pregnancy rate (bPR, hCG elevation only) and clinical pregnancy rate (cPR, gestational sac found on ultrasound), defined as number of pregnancies per embryo transfer, and implantation rate (IR), defined as number of gestational sacs per number of transferred embryos were evaluated retrospectively in the following groups:

Group I – only Pattern "0" or "other" embryos transferred, i.e. pronuclear morphology scoring

Group II – only EC or NEC embryos transferred, i.e. EC scoring

Group III – EC and Pattern "0" or "other" embryos transferred, i.e. joint scoring system

Group IV – Pattern "0" and EC or NEC embryos transferred, i.e. joint scoring system

### Statistical analysis

Statistical analysis was performed using STATISTICA *(StatSoft, Inc. 2001)*, STATISTICA CZ version 8.0 [19] and SPSS version 12.0 software. Mann-Whitney U test and t-test for independent samples and for differences between relative values were used, p < 0.05 was considered statistically significant.

## Results

Over the study period a total of 4244 oocytes in 364 IVF cycles were retrieved. Fertilization by IVF and ICSI was achieved in 3166 oocytes (overall fertilization rate 75%). Polyploidy was found in 458 zygotes. Of the 2708 normally fertilized oocytes, 754 supernumerary zygotes were cryopreserved in the PN stage and the remaining 1954 zygotes were available for retrospective analysis of the predictive value of various embryo quality scoring systems.

### Pronuclear zygote morphology (Group I)

Pattern "0" was observed in 850 zygotes (31%) and "other" pattern in 1858 zygotes (69%). Pattern "0" zygotes were transferred in 109 cycles and "other" pattern zygotes in 140 cycles regardless of their cleavage rate. Cycles where mixed pattern zygotes had to be used for transfer were not evaluated. The analysis of cycles where the selection of embryos for transfer was based on pronuclear morphology is given in Table 1. No difference in outcome parameters between pattern "0" and "other" pattern embryos was found, even though there were significantly more embryos transferred in the "other" pattern group.

**Table 1:** Outcome parameters in Group I (pronuclear zygote morphology)

Pattern of embryos transferred	**"0"**	**"Other"**	**p**
Number of cycles	109	140	
Maternal age (mean ± SD)	30.7 ± 3.9	31.3 ± 3.7	>0.5
Total number of embryos transferred	190	269	
Mean ± SD embryos/cycle	1.7 ± 0.5	1.9 ± 0.5	0.033
Biochemical pregnancies	58	74	
bPR (%)	53	53	>0.5
Clinical pregnancies	50	65	
cPR (%)	46	46	>0.5
Number of gestational sacs by US	66	78	
IR (%)	35	30	>0.5

### EC scoring (Group II)

Similarly, 599 embryos (30%) were classified as EC and 1379 (70%) as NEC. EC embryos were transferred in 120 cycles and NEC embryos in 152 cycles, regardless of their pronuclear zygote morphology scoring. Again, the remaining cycles with transfer of both EC and NEC embryos were excluded from the evaluation. The data of cycles where selection of embryos for transfer was based on cleavage rate are given in Table 2. Contrary to the pronuclear morphology scoring, clinical pregnancy rates and implantation rates were greater (p < 0.003 and p < 0.006, respectively) after transfers of EC embryos compared to NEC embryos.

**Table 2:** Outcome parameters in Group II (cleavage rate)

Cleavage rate of embryos transferred	EC embryos	NEC embryos	p
Number of cycles	120	152	
Maternal age (mean ± SD)	30.6 ± 3.6	31.4 ± 3.8	>0.5
Total number of embryos transferred	214	291	
Mean ± SD embryos/cycle	1.8 ± 0.5	1.9 ± 0.5	>0.5
Biochemical pregnancies	73	70	
bPR (%)	61	46	>0.5
Clinical pregnancies	67	58	
cPR (%)	56	38	0.003
Number of gestational sacs by US	82	77	
IR (%)	38	27	0.006

### "Joint" pronuclear morphology and EC scoring (Group III and IV)

A comparison of outcome of the combination of both scoring systems could be applied only in 137 cycles.

### Group III

There was no difference in outcome parameters between 50 cycles where only EC and Pattern "0" embryos were transferred and 48 cycles where only EC and "other" pattern embryos were transferred (Table 3). However, in the latter group more embryos per transfer had been used which could also influence the outcome.

**Table 3:** Outcome parameters in Group III (combined scoring) where only EC embryos with either pattern "0" or "other" were transferred

	EC + "0"	EC + "Other"	p
Number of cycles	50	48	
Maternal age (mean ± SD)	30.3 ± 4.0	31.6 ± 4.0	>0.5
Total number of embryos transferred	75	93	
Mean ± SD embryos/cycle	1.5 ± 0.5	1.9 ± 0.5	0.001
Biochemical pregnancies	30	28	
bPR (%)	60	58	>0.5
Clinical pregnancies	28	25	
cPR (%)	56	52	>0.5
Number of gestational sacs by US	32	31	
IR (%)	43	33	>0.5

### Group IV

In 50 cycles only Pattern "0" and EC embryos and in 39 cycles only Pattern "0" and NEC embryos were used for transfer. Significantly better outcome parameters were achieved when only Pattern "0" and EC embryos were used, in spite of the fact that, on average, less embryos were transferred (Table 4).

**Table 4:** Outcome parameters in Group IV (combined scoring) where only Pattern "0" embryos with either EC or NEC were transferred

	"0" + EC	"0" + NEC	p
Number of cycles	50	39	
Maternal age (mean ± SD)	30.3 ± 4.0	31.6 ± 3.4	>0.5
Total number of embryos transferred	75	75	
Mean ± SD embryos/cycle	1.5 ± 0.5	1.9 ± 0.5	0.000
Biochemical pregnancies	30	17	
bPR (%)	60	44	>0.5
Clinical pregnancies	28	12	
cPR (%)	56	31	0.020
Number of gestational sacs by US	32	20	
IR (%)	43	27	0.038

## Discussion

Embryo quality and implantation potential are two of the most important factors that influence the outcome of AR procedures. The contemporary tendency is to decrease the number of transferred embryos to prevent multiple pregnancies while still achieving an acceptable success rate. The ultimate goal is to transfer, whenever possible, only one top quality embryo. Therefore much effort has been invested in the search for a suitable non-invasive method of scoring embryo quality. Besides improving outcome parameters, recognizing high quality embryos may save pointless costs of cryopreserving low quality embryos, some of which may be damaged due to oxidative stress and/or other factors [20].

In the hands of an experienced embryologist, both zygote and EC scoring and morphological evaluation of embryos require only basic laboratory equipment and are therefore well suited for routine clinical use. Unfortunately, data derived from *in vitro *cultured human embryos suggest that the quality of embryos often changes from day to day. Top quality zygotes may become low quality embryos after *in vitro *cultivation and *vice versa *and with this in mind we have retrospectively evaluated the merits of these two scoring systems individually and in combination.

There is no consensus yet on the preference of these two basic scoring systems. In our earlier reports [13,14] some benefits of introducing the EC scoring systems into clinical practice were shown. Later we implemented the pronuclear zygote morphology for evaluation of embryo quality. In the group where embryos were transferred according to their pronuclear zygote morphology no difference in outcome parameters between pattern "0" and "other" pattern embryos (cPR 46% versus 46% and IR 35% versus 30%) was found, even though there were significantly more embryos transferred in the "other" pattern group. This is in line with observations by Salumets et al. [21] who did not find any significant differences in implantation and pregnancy rates between the different pronuclear zygote morphology patterns after the elective single embryo transfer. Similarly, James et al. [7] found no correlation between zygote morphology and live birth rate. On the other hand, a significant difference in IR with regard to the polarization of NPB was found by Lukaszuk et al. [8]. Nagy et al. [22] observed a strong correlation between polarization of the NPB in both pronuclei and faster cleavage when the evaluation was done earlier than usual, as little as 15 hrs after insemination. The timing of evaluation may have an important effect. It has been shown that pronuclear morphology changes over time as the number of small pronuclear precursor's declines when they merge into larger nucleoli, with polarization of NPB in both pronuclei [17,22].

Our present study confirmed the greater predictive value of the EC embryo quality scoring system as compared to pronuclear zygote morphology. Significantly better outcome parameters for clinical pregnancy rate (56% versus 38%) and for implantation rate (38% versus 27%) were achieved after transfers of EC embryos compared to NEC embryos. The possibility that early embryo cleavage, a highly significant biological indicator of embryo growth potential, may predict IVF outcome was first proposed by Shoukir et al. [3] and Sakkas et al. [5,23]. Lundin et al. [24] also showed that early cleavage was a strong independent predictor of birth, especially after ICSI procedures. In a study which included analysis of 178 elective single embryo transfers Salumets et al. [12] also showed that EC embryos possess a significantly higher developmental competence than NEC embryos. One of the mechanisms that determine the speed of embryo development may be the quality of the spermatozoon, as was suggested by Chen and Kattera [25]. The best outcome in terms of pregnancy rate (56%) and implantation rate (43%) in our study was found when only Pattern "0" and EC embryos were used for transfer in spite of the lower average number of transferred embryos (1.5). This observation is supported by Petersen et al. [11] who used similar combined criteria for embryo quality. In 36 patients where 100% implantation rate was achieved, 82% of transferred embryos had optimal zygote morphology and cleaved early.

## Conclusion

The ultimate goal of multiple pregnancy prevention in IVF is elective single embryo transfer. This trend stimulates further research in refining methods to assess embryos with the best implantation potential. Our results indicate that early cleavage is a better independent marker of implantation potential than zygote morphology. But the best outcome can be achieved if both embryo scoring systems are used jointly and the embryo is classified as EC and Pattern "0".

## Competing interests

The authors declare that they have no competing interests.

## Authors' contributions

JB designed the study, carried out all embryo evaluation and selection for transfer, provided data collection and drafted the manuscript; IO selected the stimulation protocols, provided clinical information, performed embryo transfers and coordinated the study; MS participated in the daily routine embryology; HF supervised the manuscript design, language corrections and prepared data for statistical evaluation and gave final approval. All authors read and approved the final manuscript.
